# Study protocol for a cluster randomized trial of the Community of Voices choir intervention to promote the health and well-being of diverse older adults

**DOI:** 10.1186/s12889-015-2395-9

**Published:** 2015-10-13

**Authors:** Julene K. Johnson, Anna M. Nápoles, Anita L. Stewart, Wendy B. Max, Jasmine Santoyo-Olsson, Rachel Freyre, Theresa A. Allison, Steven E. Gregorich

**Affiliations:** Institute for Health & Aging, University of California, 3333 California St., Suite 340, San Francisco, CA 94118 USA; Center for Aging in Diverse Communities, University of California, San Francisco, USA; Division of General Internal Medicine, Department of Medicine, University of California, San Francisco, USA; Division of Geriatrics, Department of Medicine, University of California, San Francisco, USA; Department of Family and Community Medicine, University of California, San Francisco, USA

**Keywords:** Health promotion, Arts, Music, Health disparities, Older adults, Translational research, Community-based participatory research

## Abstract

**Background:**

Older adults are the fastest growing segment of the United States population. There is an immediate need to identify novel, cost-effective community-based approaches that promote health and well-being for older adults, particularly those from diverse racial/ethnic and socioeconomic backgrounds. Because choral singing is multi-modal (requires cognitive, physical, and psychosocial engagement), it has the potential to improve health outcomes across several dimensions to help older adults remain active and independent. The purpose of this study is to examine the effect of a community choir program (Community of Voices) on health and well-being and to examine its costs and cost-effectiveness in a large sample of diverse, community-dwelling older adults.

**Method/design:**

In this cluster randomized controlled trial, diverse adults age 60 and older were enrolled at Administration on Aging-supported senior centers and completed baseline assessments. The senior centers were randomly assigned to either start the choir immediately (intervention group) or wait 6 months to start (control). Community of Voices is a culturally tailored choir program delivered at the senior centers by professional music conductors that reflects three components of engagement (cognitive, physical, and psychosocial). We describe the nature of the study including the cluster randomized trial study design, sampling frame, sample size calculation, methods of recruitment and assessment, and primary and secondary outcomes.

**Discussion:**

The study involves conducting a randomized trial of an intervention as delivered in “real-world” settings. The choir program was designed using a novel translational approach that integrated evidence-based research on the benefits of singing for older adults, community best practices related to community choirs for older adults, and the perspective of the participating communities. The practicality and relatively low cost of the choir intervention means it can be incorporated into a variety of community settings and adapted to diverse cultures and languages. If successful, this program will be a practical and acceptable community-based approach for promoting health and well-being of older adults.

**Trial registration:**

ClinicalTrials.gov NCT01869179 registered 9 January 2013.

## Background

The United States (US) is experiencing a rapid increase in the number of older adults, and finding ways for them to remain active, independent and involved in their communities has become a priority [1]. In 2012, there were 43.1 million individuals over age 65 in the US, and this number is expected to almost double by 2050 [2]. By 2030, nearly half of these older adults are expected to come from diverse racial/ethnic backgrounds [3]. This rapid rise in the proportion of diverse older adults will significantly increase health care costs because, relative to non-minority elders, they are at increased risk of poor health outcomes [4–7]. Thus, there is an immediate need to identify novel, cost-effective approaches that promote health, independence, and well-being and can be applied to an expanding and increasingly diverse population of older adults.

Music, being an integral part of most cultures, offers one such possibility for engagement. Active participation in music (e.g., playing musical instruments or singing in groups) may have positive health benefits for older adults [8–13]. Of the participatory music traditions in the US, group singing requires the fewest prerequisites in terms of musical skill or training. With singing, the musical instrument is located within the body, and basic singing abilities are thought to develop spontaneously in young children without formal music training [14]. In the US, choral singing is the most popular creative arts activity with approximately 32.5 million adults regularly singing in 270,000 choirs [15]. Community choirs or other non-professional choral groups typically draw members from the community at large and include individuals with a wide range of musical experience and abilities [16].

Singing in a choir offers several advantages. Group singing has the benefit of being social, as well as requiring cognitive and physical engagement. Studies suggest that activities involving such a combination of cognitive, psychosocial and physical components may confer additional health benefits over activities that involve only one component [17–19]. Because of this multi-modal characteristic of choral singing, by potentially improving psychosocial, cognitive, and physical aspects of health, it could in turn help older adults remain active and independent. Last, group singing can be done throughout the life course, thus providing an activity that can be sustained regardless of declining functioning due to age. Taken together, community choir singing is a potentially appealing activity for older adults and may confer multiple benefits to health and well-being.

A growing body of literature suggests a positive association between choral singing and health and well-being for older adults. Several cross-sectional studies found that adults who sing in a community choir tend to endorse high ratings of well-being and mood [20–24]. However, cross-sectional studies are not able to determine causality, and the majority of studies to date involve persons of higher socioeconomic status (SES). What remains unclear is whether these effects can be causally attributed to engaging in choral singing or to the self-selection of the participants. Longitudinal and randomized controlled studies are needed to address these questions.

To date, three longitudinal intervention studies of choral singing for older adults have been completed. Cohen and colleagues (2006) were the first to initiate a large-scale non-randomized study comparing 12 months of participation in a community choir for older adults to a usual activity comparison group. The choir met weekly for 33 weeks over a one-year period. At 12 months (85 % of those enrolled completed the 12 month assessment), those in the choir group reported higher self-rated health, fewer doctor visits, fewer over-the-counter medications, fewer falls, and less decline in morale and loneliness than the comparison group [25]. However, the groups were self-selected (recruited separately into the singing and comparison group), bias due to attrition was not considered, and the sample consisted predominately of non-Latino White women. Nonetheless, improvement in measures of several domains of health and well-being suggested better outcomes for the choir group, an important finding given the lack of prior studies. More recently, Coulton and colleagues completed a randomized controlled trial of a singing program for older adults in England [26]. Adults age 60 and older recruited from senior centers and nearby communities were randomized to a standardized singing program or a usual activity control group. The 90-min singing group met weekly for 14 weeks. There were significant differences between the intervention and control group after three months; participants in the singing group reported significantly higher scores on a mental health summary index and lower depression and anxiety, compared with the control group. Although the participants were randomized to the intervention and control groups, the sample was, again, primarily White women with high educational backgrounds. The outcomes focused only on mental health; thus the possible effects on other health aspects were not evaluated. The third study was a randomized controlled trial of a theater program for lower-income older adults that included a singing group as an active control condition, in addition to a usual-activity control group [27]. However, the authors did not report differences between the two control groups (e.g., between the usual activity group and the singing group). Within-group analyses indicated that the singing group improved on ratings of personal growth over the 4 weeks (p < .001). No other within-group changes were examined for significance.

Considering all of the evidence and because choral singing can involve cognitive, physical and psychosocial engagement, community choirs could help older adults remain active and engaged. In addition, community choir programs can be culturally tailored to increase the reach to an expanding and increasingly diverse population of older adults. Community choirs are thus a promising strategy for addressing some of the health disparities in older adults. We aimed to evaluate the benefits of community choir singing across a variety of health domains in a sample of ethnically and socioeconomically diverse older adults.

The purpose of this manuscript is to describe the study protocol for this recently implemented cluster randomized trial of a community choir program (Community of Voices) delivered within senior centers to promote health and well-being of diverse older adults. The study was designed to overcome some of the limitations of the prior studies. Specifically, we included diverse older adults, we took a comprehensive approach to measuring health and well-being outcomes with a focus on cognition, physical function, and psychosocial variables, and we aimed to recruit a large sample to address statistical power concerns through a partnership with a local Administration on Aging (AoA).

## Method/design

### Overall study design

The Community of Voices study is a multi-site, cluster randomized trial, located in San Francisco, CA (study enrollment from February 2012 – August 2015). Twelve AoA- supported senior centers were randomized to receive the 12-month Community of Voices choir program immediately (intervention group) or after a 6-month delay (wait-list control group). Adults age 60 or older were recruited from the geographic service areas of each senior center. Recruitment and enrollment were conducted in staggered pairs of centers. We completed a comprehensive battery of outcome measures, including cognitive, physical, and psychosocial outcomes hypothesized to be mechanistically related to choral singing. We assessed primary and secondary outcomes for all participants at baseline, 6 and 12 months. Thus, for those in the control group, the study lasted 18 months (6 month wait period, 12- month choir). The main randomized comparison will be at 6 months. The 12-month assessment allows us to examine within-person change over the 12- month period of the choir.

### Study aims

The main study aims were twofold: 1) to examine the effect of the Community of Voices program on health and well-being and 2) to examine its cost-effectiveness in terms of quality adjusted life years (QALYs). We also assessed program costs, including cost/person served.

### Human subjects

The study protocol, consent forms, and outcome measures were approved by the University of California, San Francisco’s Committee on Human Research. All participants signed and were given a copy of the consent form along with a copy of the Bill of Rights for Research Participants before beginning the study.

### Establishing partnerships and collaborations

We began by contacting the local AoA (Department of Aging and Adult Services or DAAS of San Francisco County) to discuss the possibility of conducting a study to examine the effects of community choirs on the health and well-being of diverse older adults. Given their interest, DAAS became a collaborator at the outset. DAAS administers a large network of senior and activity centers that serve older adults of diverse ethnic/racial and SES backgrounds. All centers offer a range of activities, typically provided by bilingual staff. We then identified all AoA-supported senior centers in San Francisco county that served older adults using the DAAS website (N = 32). We contacted senior center directors and discussed their interest in the study, availability of older adults who spoke English or Spanish (including monolingual Spanish speakers), and the availability of space for weekly choir sessions and private rooms for study assessments. Sixteen senior centers meeting these criteria were initially approached, and the first 12 senior centers to agree were included. For the senior center directors who were interested in the study, we also discussed and received feedback about the research questions, study design, and the choir program.

During the course of the study and before recruitment was started or randomization was revealed, two senior centers withdrew from the study due to changes in physical resources that limited their ability to host the study. Two additional senior centers with similar demographic characteristics to these two centers were identified, approached and agreed to participate in the study.

To identify community best practices in the initial stages, we partnered with the San Francisco Community Music Center, a local non-profit, community music organization with experience delivering music programs for diverse and low SES residents of all ages. The music center was then directing an older adult choir in a senior center in San Francisco. In addition, the principal investigator (PI) had spent six months studying older adult choirs in Finland, a country that promotes lifelong participation in community choirs [21].

### Recruitment approach

To recruit older adults from each senior center, our sampling frame included those already attending each senior center and residents from neighborhoods in geographic service areas in close proximity to the senior centers. This strategy was based on our previous work recruiting diverse, lower SES individuals [28]. We used multiple recruitment methods, such as traditional media (e.g., flyers, postcards, community television, and radio) as well as presentations at senior centers and nearby senior housing. We also developed a large network of community organizations involved in aging services (e.g., community health clinics, social workers, aging services) and shared recruitment materials with them to distribute to older adults they served. Word of mouth was also an effective recruitment approach as those who received our recruitment materials in turn shared the news of the study with their friends and family.

Through these outreach methods, interested individuals were invited to attend an informational meeting about the study at the senior centers or to discuss the study over the phone with a staff person. At the meetings or by phone, the choir intervention content, duration, study procedures, risks, and randomization procedures were explained in English or Spanish and questions answered. For those who attended an informational meeting and wished to enroll, a screening assessment was completed at the end of the presentation or was scheduled at a later date at the same center. For those reached by phone who expressed interest, most of the screening was done by the staff person on the phone; any remaining screening items (e.g., cognitive testing) were done at the subsequent enrollment visit.

### Participant inclusion/exclusion criteria

Inclusion criteria included being age 60 and older, having adequate visual and hearing acuity (with assistive devices), and English or Spanish fluency (including bilingual and monolingual Spanish speakers) to complete the study assessments.

Exclusion criteria included having significant cognitive impairment or a diagnosis of dementia (e.g., Alzheimer disease), having an unstable or serious medical condition that would limit participation in weekly choir sessions or assessments, or plans to move out of the area within 12 months. Potential participants who were currently singing in a choir, defined as regularly singing in a choir during the past six months (e.g., weekly) were also excluded. For example, someone singing in a choir that met weekly (organized singing group) was excluded, but someone who sang weekly as part of typical activities, like attending a worship service and singing along with the hymns, was not excluded.

### Screening measures

Screening for inclusion and exclusion criteria was done in person at each of the senior centers either at the informational meeting or at a special screening session. With verbal consent, potential participants were asked a series of questions related to inclusion/exclusion criteria. If a particular answer indicated they were not eligible, they were told they were not eligible and given the reason. For these individuals, staff explained that the alternative to participating in the study included the opportunity to join other choirs in the community, and they were given a list of these other community choirs in the area. However, all individuals completed all demographic questions (first 7 questions) before being told of being ineligible.

To determine whether a potential participant had the language skills necessary to complete the study assessments in either English or Spanish, we administered two questions: individuals were asked to rate both their English and Spanish fluency using a 5-point scale (not at all, poorly, fairly well, well, or very well) [29]. Those who rated their fluency in either language as fairly well, well, or very well met the inclusion criterion.

We ascertained the presence of significant cognitive impairment or dementia by self-report of a physician diagnosis and a cognitive screening test. If the individual reported a physician diagnosis, the screening interview was ended (ineligible). If not, we administered the Mini-Cog [30], a screening test for identifying clinically-significant cognitive impairment available in both English and Spanish [30]. It has also been validated in diverse older adult populations [31]. The Mini-Cog involves a three-item word recall and clock drawing. We included individuals who could recall at least two of the three words after the short delay.

To assess adequacy of visual and auditory acuity (with correction) to complete the study assessments, the study staff made a determination based on their interaction with the individual (whether in person or by phone). If there was any uncertainty on the study staff’s behalf, individuals were asked to rate their visual and hearing acuity on a 3-point scale (poor, fair, good); those reporting poor visual or hearing acuity were excluded.

We ascertained serious medical conditions by asking whether they had a current serious medical or mental health condition that might limit their ability to take part in the assessments or weekly choir. We excluded those with self-reported current (but not prior) severe medical or mental health conditions. We asked whether they had plans to move out of the area within 12 months; and if so, they were not eligible. We asked if they had regularly sung in a choir in the past 6 months, and excluded those who reported singing weekly (those singing less than weekly or not at all were eligible).

### Cluster randomization

Because of the small number of centers involved, simple random assignment of centers to the immediate and delayed start groups could fail to establish equivalent experimental groups at baseline. Therefore, we used restricted randomization (described below) to help ensure balance of center characteristics across experimental groups [32]. We collected data from senior center reports that described the characteristics of each center, including individual-level variables, center-level variables, and readiness for the intervention (Table 1). These variables were used to aid the selection of two matched groups of centers, with each group containing six centers. An algorithm generated all possible partitions of centers into two groups of six centers (462 possible partitions), estimated the profile of center characteristics for each group, and calculated the sum of squared profile differences across groups within each partition. We examined 23 (top 5 %) candidate partitions with the smallest sum of squared profile differences and selected one partition based upon visual inspection of the group-specific profiles. Because recruitment was conducted within staggered pairs of centers, six pairs of centers were created, and each pair included one center from each group. Finally, one group of six centers was randomized to the immediate start group and the other set to the wait-list control group. When recruitment was completed within a staggered pair of senior centers, the randomization assignment was revealed to the corresponding study staff, senior centers, and participants.

**Table 1:** Variables used in the restricted randomization process

Level	Variables
Individual level	% needing translation services
	% living alone
	% functionally impaired
	% with “low” income
	% with medical insurance
	% on social security income
	% with nutrition risk
	% age < 60
	% age 60-74
	% age 75-84
	% age 85+
	% African American
	% Asian/Pacific Islander
	% Latino
	% White
	% male
Center level	Whether the following types of activities are offered:
	• cognitive
	• emotional
	• singing
	• arts/crafts
	• health promotion
	• meal service
	• social services
	• transportation services
	• parties/cultural events
	• day trips
Readiness for intervention	Moderately, quite, or extremely

### Intervention: community of voices choir program

The Community of Voices (Comunidad de Voces in Spanish) choir program was designed specifically with the goal of promoting health and well-being of diverse, community-dwelling older adults. Details about the choir intervention are reported in a separate manuscript. Here, we briefly summarize the intervention.

The program was designed using a translational research approach [33] that incorporated information from evidence-based research on the benefits of singing for older adults, community best practices related to community choirs for older adults, input from experts in delivering choral programs in community settings, and the perspective of senior center directors. The literature was summarized in the introduction. The San Francisco Community Music Center helped design the program. The PI observed an older adult choir directed by the music center and discussed the development with music professionals at the center. Other best practices were considered, such as the older adult choirs that the PI observed during her research in Finland on choral singing [21].

The program was designed to be led by professional choir conductors and accompanists. Professional musicians, some of whom were bilingual, were used to help assure a high level of standardization of program elements [25]. The music center staff was responsible for helping hire, train, and supervise the choir conductors and accompanists. The conductors and accompanists completed training on program components before beginning the choir, and refresher training was conducted as needed.

The content of the choir program was based on literature suggesting that activities involving a combination of cognitive, physical, and psychosocial components may confer additional health benefits over activities that involve only one element or are performed singly [17–19]. The program content targeted three components hypothesized to be the primary pathways by which the choir program promotes health and well-being for older adults: cognitive, physical and psychosocial engagement. To provide cognitive engagement, the choir sessions involved different strategies to learn new songs (e.g., aural learning of separate parts, singing from written song lyrics or musical notation, call and response methods), review of previously learned songs, practice listening to fellow singers, attending to the choir conductor, and synchronizing personal singing parts with the rest of the choir. To provide physical engagement, the sessions included a combination of sitting and standing, moving to different parts of the room to sing, discussion by the choir director about body posture and breathing, and focus on the use of abdominal and chest muscles involved in breathing. To provide psychosocial engagement, the sessions included working toward a common goal with others, exercises to promote group cohesion, a 10-min break for refreshments and socialization, and discussion of the meaning of the songs and their cultural history. Each choir session included activities related to these three components. In addition, the choirs participated in 3-4 informal, public performances. The approach to the choral program across sites was standardized around these components, which were documented in a manual. The music center was responsible for helping assure that the choir program components were consistently implemented. Additional fidelity checks were done by the researchers (described below).

All choir sessions took place at the participating senior centers. Each choir met once a week for one year (44 weeks with breaks occurring during holidays); the sessions were 90 min in length with a 10-min break for snacks. A 15-min period at the beginning of each session was used as a warm-up and included vocal exercises, breathing exercises, standing, and stretching exercises.

The overall music style (e.g., Latin folk music, show tunes, traditional African American music, and Filipino folk music) for each choir was selected by the music center after meeting with each senior center director and considering the cultural backgrounds and music preferences of the older adults they served. The repertoire was further refined in terms of cultural relevance and complexity during the first several weeks after the start of each choir, and requests for specific songs were considered. The repertoire was selected to be appropriate for older adults with a range of singing abilities and prior experience with choral singing (from beginner to advanced levels).

### Study outcomes and data collection methods

The primary and secondary outcomes were selected based on the hypothesized effects of the cognitive, physical, and psychosocial engagement components of choral singing. Table 2 lists specific components that were targeted during the choir intervention, their hypothesized links to the study outcomes, and the specific outcome measures assessed.

**Table 2:** Components of community of voices intervention, hypothesized mechanisms of action, outcomes, and specific outcome measures

Component	Mechanism(s)	Outcomes	Specific outcome measures
COGNITIVE ENGAGEMENT			
Attend to conductor, music, and fellow singers; being flexible, organizing materials	Cognitive stimulation, brain	Attention/executive function	• *Trailmaking test*
			• NIH Toolbox Flanker
Learn and recall new music (lyrics, melody, pitch, and rhythm)	Cognitive stimulation, brain	Verbal learning and memory	• NIH Toolbox Rey Auditory Verbal Learning test
PHYSICAL ENGAGEMENT			
Stand and sit, move to rhythm of songs	Balance, body strengthening	Lower body strength, balance, falls	• *SPPB Chair stands*
			• NIH Toolbox Standing Balance
			• Self-reported falls
Stand and sit, move to different parts of room, breathe deeply to sing	Stamina	Walking speed	• NIH Toolbox Gait Speed
PSYCHOSOCIAL ENGAGEMENT			
Singing feels good, is uplifting, is intrinsically pleasurable and emotionally meaningful	Reduce depressive symptoms and anxiety, increase positive emotions	Emotional well-being	• PHQ-8
			• NIH Toolbox Sadness
			• NIH Toolbox Positive Affect
			• NIH Toolbox Fear Affect
Build social network, make new friends	Increase sense of belonging and social support, decrease feelings of loneliness	Social support, loneliness	• NIH Toolbox Loneliness
			• MOS Social Support
Somewhere to go, regular activity	Something interesting to do	Interest in daily life	• NIH Toolbox Apathy
Taking on new challenges, mastering new skills with practice over time	Improve sense of mastery, increase confidence	Self-efficacy	• NIH Toolbox Self-Efficacy

Primary outcomes are noted with italicized font; all other outcome measures are secondary

All primary and secondary outcomes were assessed at baseline, 6-months, and 12-months. For the convenience of participants, all assessments, including screening, took place at the senior center in which the person was enrolled. We collected data about falls and health care service utilization every three months either during the in-person assessments or by phone in between in-person assessments.

We used the National Institutes of Health (NIH) Assessment Center^SM^ as a platform to collect and manage all data (https://www.assessmentcenter.net). This is a free, secure, online data collection tool that provides real-time scoring of measures, automated accrual reports, and real-time data export. The Assessment Center enables researchers to create study-specific websites for capturing participant data securely online. Studies can include measures within the Assessment Center library such as the NIH Toolbox measures, as well as custom instruments entered by the researcher. We used the NIH Toolbox for the Assessment of Neurological and Behavioral Function [34] to assess many of the outcomes. The NIH Toolbox primarily uses a computer adapted testing (CAT) format based on item response theory (IRT); short-forms are also available which provide a fixed number of items. All NIH Toolbox measures are available in English and Spanish. We added several custom instruments that were not part of the Toolbox; all non-Toolbox measures were customized to be included as part of the Assessment center platform (e.g., format, randomization, skip patterns). All cognitive and physical function measures were performance based (e.g., required participants to perform a task such as walking or recalling words). All other measures were interviewer-administered. Below, we describe our primary and secondary outcomes including the rationale and specific measure used for each variable.

#### Primary outcomes

Our three primary outcomes include one from each of the hypothesized mechanisms of action of the intervention: cognitive, physical, and psychosocial engagement.

##### Cognitive engagement: attention/executive function

Attention and executive function refer to cognitive processes that require selective focusing, storing, and manipulation of information. We used two measures of attention/executive function, one of which was a primary outcome and one a secondary outcome. For the primary outcome measure, we used the Trail Making Test (TMT) [35, 36] which is a commonly used test of the set-shifting aspect of executive function. Studies involving music instrument training for older adults found improvement in executive function [37]. The TMT has been used as an outcome measure in clinical trials with community-dwelling older adults [38], including those from diverse and lower SES backgrounds [39, 40]. The TMT has two conditions: condition A requires participants to connect numbers in order as quickly as possible, while condition B requires participants to connect numbers and letters, alternating in order, as quickly as possible. Time to complete each condition is the variable of interest. We used Trails condition B minus Trails condition A (time) as an index of executive function because it isolates the executive control component of the TMT [41].

##### Physical engagement: lower body strength

Our primary outcome measure of physical engagement focused on lower body strength. We used the chair stands measure from the Short Physical Performance Battery (SPPB) [42] which is widely used for evaluating lower body strength in older adults. A previous study found that choral singing was associated with fewer falls [25], which may be related to improvement in lower body strength. Poor performance on the chair stands task is associated with poor health outcomes, such as functional decline, hospitalizations, and mortality [42, 43]. Chair stands have been used in several community-based clinical trials of older adults [44–47], some of which have included diverse individuals. The time to complete 5 chair stands was the primary variable of interest.

##### Psychosocial engagement: emotional well-being

We used several measures of emotional well-being, one of which was a primary outcome. The 8-item Patient Health Questionnaire (PHQ-8) [48] was used to assess depressive symptoms as the primary psychosocial outcome. In a previous study with a choir intervention for older adults, Cohen and colleagues found a trend for improvement in depressive symptoms [25]. The PHQ-8 asks participants to rate the frequency of depressive symptoms in the last two weeks including feeling depressed, having little interest in things, having trouble sleeping, being tired, having poor appetite, feeling bad about oneself, having trouble concentrating, and moving slowly. The PHQ-8 has good validity and reliability in diverse community samples [49, 50].

#### Secondary outcomes

All other outcome measures shown in Table 1 are secondary outcomes.

##### Cognitive engagement: attention/executive function

We used the NIH Toolbox Flanker Inhibitory Control and Attention Test as a secondary outcome measure of executive function. This measure taps both attention and inhibitory control. It is considered a “fluid ability,” assessing the capacity for new learning and information processing in novel situations. The test requires participants to focus on a given stimulus (a central arrow) when flanked by either congruent (same direction) or non-congruent (different direction) arrows. Participants complete 20 trials, and the final score reflects a combination of accuracy and reaction time over the 20 trials.

##### Cognitive engagement: verbal learning and memory

We used the NIH Toolbox Rey Auditory Verbal Learning Test as a secondary outcome of cognitive function. This is a word-list measure of “episodic memory” in which 15 unrelated words are presented orally (via audio recording) over three consecutive trials. After each presentation, participants were asked to recall as many words as possible. The measure is the sum of correct words for all three trials (range 0-45). We expanded this test to include two additional measures. After the three trials, participants listened to a (distractor) list of 15 new words and were asked to recall as many as they could (learning distractor list, score 0-15). Immediately after the distractor list, participants were asked to recall as many of the original 15 words as possible (short-term recall, score 0-15). The distractor and delayed recall conditions modifications were suggested by Dan Mungas (personal communication).

##### Physical engagement: balance and falls

As secondary outcome measures of physical engagement, we assessed balance and falls. The NIH Toolbox Standing Balance measure assesses static standing balance. Participants assume and maintain 5 poses. Four involve standing with their feet side-by-side (together) with these variations: eyes open on solid surface, eyes closed on solid surface, eyes open on foam surface, and eyes closed on foam surface. The fifth pose is to stand tandem (one foot aligned in front of the other) on a solid surface with eyes open. Participants were asked to hold each position for 50 s and postural sway is recorded for each pose using an accelerometer worn by the participant. Scoring involves their ability to hold the position for the time specified and the postural sway. We used the two scores recommended by the Assessment Center from these data: 1) ratio of poses 2/1 reflects the ability to use input from somatosensory and vestibular systems to maintain balance, and 2) ratio of poses 4/1 reflects the relative reduction in postural stability when visual and somatosensory inputs are simultaneously disrupted (typically representative of the effectiveness of vestibular function for postural control).

To measure falls, we assessed the frequency of falls over the past 3 months using a single question commonly used in several large studies about falls [51, 52]. The question was asked at 3-month intervals to obtain a complete assessment of falls over the 12 month period.

##### Physical engagement: walking speed

To assess walking speed, we selected the NIH Toolbox performance measure of gait speed which is a measure of “walking pace”. This is a timed walk in which participants were asked to walk 4 meters at their usual pace. They completed two timed trials with the score on the fastest trial considered as the outcome (meters per second).

##### Psychosocial engagement: emotional well-being

As secondary outcomes, we included 3 additional measures of emotional well-being. The NIH Toolbox Sadness CAT measure assesses the frequency in the past 7 days the individual felt, e.g., worthless, helpless, sad, depressed, unhappy, hopeless, had nothing to look forward to, and felt like a failure. The NIH Toolbox Positive Affect CAT measure assesses the frequency in the past 7 days the person felt, e.g., cheerful, attentive, delighted, happy, joyful, enthusiastic interested, peaceful, good natured, useful, understood, content, and liked oneself. The NIH Toolbox Fear/Affect Short Form measure assesses the frequency in the past 7 days the individual felt fearful, anxious, worried, nervous, uneasy, tense, and found it hard to focus.

##### Psychosocial engagement: social support and loneliness

To assess social support we selected six items from the Medical Outcomes Study (MOS) Social Support Survey [53]. Three items each were chosen from the Positive Social Interaction Scale and from the Emotional/Informational Support scale. Items assessed the extent to which respondents had someone to: have a good time with, get together with for relaxation, do something fun with, listen when you need to talk, give you advice if needed, and who understands your problems. We also administered the NIH Toolbox Loneliness Short Form measure which determines the frequency in the past month the person felt alone, apart from others, left out, lonely, and no longer felt close to anyone.

##### Psychosocial engagement: interest in daily life

We selected the NIH Toolbox Apathy Short Form measure to assess interest in life. This asks the frequency in the past month the person felt interested in things, got things done, saw a job through, got things started on one’s own, did interesting things, and was motivated.

##### Psychosocial engagement: self-efficacy

The NIH Toolbox Self-Efficacy Short Form measure assesses one’s confidence in handling problems.

### Measures of fidelity and compliance to choir intervention

The fidelity of the Community of Voices program across senior center sites, choir conductors, and styles of music was tracked using a 16-item survey with a 4-point scale. The fidelity survey focused on (i) implementation of the three key components of the choir program (cognitive, physical, and psychosocial engagement), (ii) leadership/communication skills, and (iii) musicianship. For each item, a score of 4 indicated that the performance of the conductor/accompanist exceeded program expectations; 3 indicated that the item met program expectations, 2 indicated that it was below expectations, and 1 indicated that it was not addressed and was well below expectations. The PI completed the fidelity check every three months, with an additional assessment three weeks after each choir began. Feedback was provided to conductors if the fidelity ratings on any of the 16 items fell below 3.

To assess compliance to the intervention, attendance was recorded at each choir session by the choir directors, and research staff coded absences according to the reason for the absence (e.g., illness, medical appointment, vacation, family issues).

### Intervention cost and cost effectiveness measures

In addition to evaluating the effectiveness of the intervention, we conducted cost-effectiveness analyses (CEA) of the program. As is commonly done for CEA, a single measure of health-related quality of life was used to measure the effect of the program. In addition, we determined the cost of the Community of Voices program, including the cost of the choirs themselves, in addition to any costs associated with changes in healthcare utilization on the part of the participants. The measures of cost and effectiveness are then combined into a measure of cost-effectiveness.

#### Health-related quality of life

Because of its widespread use in clinical trials and cost-effectiveness studies, we used the EuroQol Group’s EQ-5D (a brief multi-attribute, preference-based health status measure) [54, 55] to measure health-related quality of life. It has both English and Spanish versions and can be self- or telephone-administered by an interviewer. It covers five dimensions of health: mobility, self-care, usual activities, pain/discomfort, and anxiety/depression. These dimensions can be converted to a single index value by weighting each of the response levels, and this can be used to calculate quality-adjusted life years (QALYs) for the cost-effectiveness analysis. The scale has been used with diverse older adults in the US [56–58].

#### Costs

Costs included two components: the cost of the choir program and costs associated with differences in health care utilization. All costs will be converted to 2015 dollars using an appropriate price index for the cost component (e.g. the consumer price index for supplies, a wage index for salaries, and a medical cost index for healthcare costs).

#### Choir cost

For each choir, we collected all costs related to personnel (i.e., choir conductor and accompanist salaries), supplies (e.g., music binders, file box for music, piano purchase), and services (e.g., piano tuning). We also included the cost of providing snacks during the sessions.

#### Health care utilization

We tracked utilization of health care services using a modified measure from the Chronic Disease Self-Management study [59]. Every three months, we assessed visits to a doctor, mental health provider (e.g., counselor, psychologist), other health providers (e.g., home health nurse, physical therapist), emergency room visits and hospitalizations (including number of nights hospitalized and reason for stay), and outpatient surgeries over the past 3 months. The cost per unit of utilization (hospital days) was obtained from the Medical Expenditures Panel Survey and applied to the number of units used to obtain healthcare costs.

#### Cost-effectiveness analysis

The incremental cost-effectiveness ratio (ICER) is calculated as the difference in cost divided by the difference in QALYs for the immediate start choir group compared to the wait-list control group:

$$ \mathrm{ICER}\kern0.5em  = \left({\mathrm{C}}_{\mathrm{i}}\kern0.5em \hbox{--} \kern0.75em {\mathrm{C}}_{\mathrm{c}}\right)\ /\ \left({\mathrm{E}}_{\mathrm{i}}\kern0.5em \hbox{--}\ {\mathrm{E}}_{\mathrm{c}}\right) $$

Where C = cost measured in dollars

E = effectiveness measured in QALYs

i = the intervention (immediate start choir) group

c = the wait-list control group

The ICER is the cost per QALY resulting from the program, and can be compared to other interventions. Interventions are typically considered to be reasonable if the cost per QALY is $50,000 - $100,000 or less.

### Statistical analysis plan

Descriptive analyses will estimate means and proportions, measures of variability, and confidence intervals of all measures. When defensible and part of the best available analysis strategy, observed distributions may prompt normalizing transformations. We will determine whether participant baseline characteristics are independent of intervention group assignment (randomization check) and attrition (attrition analysis). Case-wise deletion and other ad hoc approaches to handling missing data will not be used for analysis because they rely on relatively strong assumptions and entail a loss of statistical power. Instead, multiple imputation will allow models to be fit to all available data and will invoke the relatively mild assumption that the data are *missing at random*, conditional on modeled variables [60–63].

Primary analyses will include fitting regression models of continuous participant outcomes. The data has a 3-level structure (centers, participants, repeated measures), which will be accommodated with linear mixed models, including random intercepts both for centers and participants. The main study end-point will be the 6-month assessment. The modeled primary outcomes include the baseline and 6-month assessments of the primary physical, emotional, and cognitive variables (see Table 2). Each outcome will be regressed onto indicators of intervention group assignment and categorical time, as well as the group-by-time interaction. Significant intervention group main effects at 6-months and group-by-time interaction effects would be interpreted and described.

Secondary analyses include models of secondary outcomes that are conceptually similar to the models described above. We will also examine within-group comparisons of 6- and 12-month outcomes to determine any added benefits of extended choral activity and compare the effect of choral participation in the intervention group to that observed in the original control group after choral group participation is offered to them. We will also explore potential intervention effect moderators by fitting models that include key interaction terms; primarily, our focus will be on interactions between experimental group with participant sex, age, and race/ethnicity. The longitudinal models described above will be refit with the inclusion of 3-way interactions (e.g., experimental group-by-sex-by-time).

#### Sample size calculations and power

We aimed to recruit 350 participants, approximately 30 from each of 12 senior centers. Assumptions included intention-to-treat analyses, 80 % power, two-tailed alpha = 0.05, 90 % retention at 6 months, unit-standardized continuous outcomes, and a linear mixed model of group differences at 6 months. Based upon published data from a study involving older adults who completed a year-long choir program [25], the largest intra-cluster correlation (ICC) for any outcome equaled 0.015. Accounting for ICC, cluster size, and attrition, the minimum detectable group difference, *d*, equaled 0.37, which compares favorably to effect sizes based upon the results reported by Cohen and colleagues [25]: overall health rating (0.41); depression (0.38); number of weekly activities (0.57 and 0.77).

## Discussion

Our study and the choir intervention are unique in several ways. First, we involved several community-based organizations in the design of both the study and intervention. Early in the process, we discussed and received feedback from the community about the research questions, study design, and community choir program by both experts in delivering services to older adults and music professionals. By following this principle of community-based participatory research, the choir program was designed to be inherently responsive to the needs of older adults and to reflect the cultural and other values of the participating community organizations.

By conducting this study in existing AoA-supported senior centers, at its conclusion, the intervention will already be embedded in these settings; thus it will be one step closer to large-scale implementation. The project also supports the value of interdisciplinary collaborations between different types of community-based organizations (e.g., senior centers and community music programs). Such collaborations can help build new relationships between sectors that do not traditionally work together. By engaging a community music center, it may be in a position to continue to offer the choir program under its auspices. Community music centers often have a cadre of trained choir directors and accompanists, and can thus provide some of the needed infrastructure.

Our study is one of the first to design and test the effect of an arts-based intervention for older adults study that specifically focuses on improving multiple, key domains of health and well-being relevant to older adults (i.e., cognitive health, physical functioning, emotional well-being, and social connectedness). We systematically designed the intervention to specifically involve components that would directly impact these multi-dimensional set of outcomes. Thus, the Community of Voices program involves three important areas of engagement (i.e., cognitive, physical and psychosocial). This is much more engaging than a typical sing-along approach to group singing for older adults.

Another important aspect of the study is that we aimed to recruit a large number of ethnically diverse older adults to address health disparities among older adults. Because diverse older adults are projected to account for a large proportion of older adults in the next several decades [3], it is important to develop health promotion programs that can not only reach them but also be easily adapted for a variety of cultures and languages. Community choirs are well-suited for addressing disparities because they can be maximally tailored to accommodate cultural backgrounds. Further, any one choir can be multi-cultural in composition; thus the music can potentially be used to increase awareness of different cultural traditions and cross racial/ethnic barriers. If successful, this trial will add to possible interventions that can help eliminate or reduce some health disparities among older adults.

The Community of Voices program has potential for widespread scalability because it is practical and relatively low-cost to deliver and administer. The nature of the choir is such that it can be folded into an existing activities schedule at senior centers with virtually no additional infrastructure. All that is required is a room to hold sessions (e.g., an activities room or auditorium) and a professional choir director/accompanist who can deliver high quality choral music. The choir can be an additional activity or lifelong learning opportunity that can be offered by many types of community-based organizations serving older adults (e.g., senior centers, community music centers, assisted living facilities) to help older adults remain healthy, independent and engaged, in addition to more traditional health-promotion activities such as exercise and nutrition programs. Thus, choral singing may have the potential to be easily translated into a number of community settings and reach a large number of diverse older adults.

Our study design reflects some methodological considerations. First, we decided to use an intervention and wait-list control condition; we were not able to add a third “active control” arm to the study due to cost limitations. We were also not able to blind the persons conducting the assessments, as the location of the assessment in specific senior centers signaled the arm of the study, and the costs to employ both study coordinators and testers were prohibitive. We decided to perform the assessments in the senior centers to maximize the convenience for the study participants, particularly for those from diverse backgrounds where traveling to a large academic research center might be a barrier. One way to get around this barrier would be to set up temporary assessment sites (not associated with the senior center) in the community and arrange for transportation, but this would also take additional resources. To reduce risk of bias, all study personnel were not made aware of the randomization assignment before it was revealed, and we will not examine any longitudinal data before all sites complete their 6-month randomized arm of the study.

In conclusion, the study is an example of a novel translational approach to address health disparities in which multiple inputs were integrated to create an intervention (evidence-based research, best practices, and the perspective of the communities in which the intervention is to be offered). This translational approach also enabled a rigorous scientific design (randomized controlled trial) to be applied to an intervention delivered in existing community settings. If the Community of Voices program is successful, it will provide an example of how encouraging participation in the creative arts (including singing) could be a new strategy for promoting health and well-being of older adults through community-based programs.

## Abbreviations

AoA: Administration on Aging
CAT: Computer adapted testing
CEA: Cost effectiveness analysis
DAAS: Department of Aging and Adult Services
EQ-5D: EuroQol five dimensions questionnaire
ICER: Incremental cost-effectiveness ratio
ICC: Intra-cluster correlation
IRT: Item response theory
NIH: National Institutes of Health
PHQ-8: 8-item patient health questionnaire
PI: Principal investigator
SES: Socioeconomic status
SPPB: Short Physical Performance Battery
QALYs: Quality adjusted life years
TMT: Trail making test
US: United States
